# Late 3d Metal-Catalyzed (Cross-) Dimerization of Terminal and Internal Alkynes

**DOI:** 10.3389/fchem.2021.635826

**Published:** 2021-03-11

**Authors:** Sebastian M. Weber, Gerhard Hilt

**Affiliations:** ^1^ Fachbereich Chemie, Philipps-Universität Marburg, Marburg, Germany; ^2^ Institut für Chemie, Carl Von Ossietzky Universität Oldenburg, Oldenburg, Germany

**Keywords:** 1,3-enynes, alkynes, dimerization, hydroalkynylation, iron, cobalt, nickel, regioselectivity

## Abstract

This review will outline the recent advances in chemo-, regio-, and stereoselective (cross-) dimerization of terminal alkynes to generate 1,3-enynes using different types of iron and cobalt catalysts with altering oxidation states of the active species. In general, the used ligands have a crucial effect on the stereoselectivity of the reaction; e.g., bidentate phosphine ligands in cobalt catalysts can generate the *E*-configured *head-to-head* dimerization product, while tridentate phosphine ligands can generate either the *Z*-configured *head-to-head* dimerization product or the branched *head-to-tail* isomer. Furthermore, the hydroalkynylation of silyl-substituted acetylenes as donors to internal alkynes as acceptors will be discussed using cobalt and nickel catalysts.

## 1 Introduction

**FIGURE 1:**
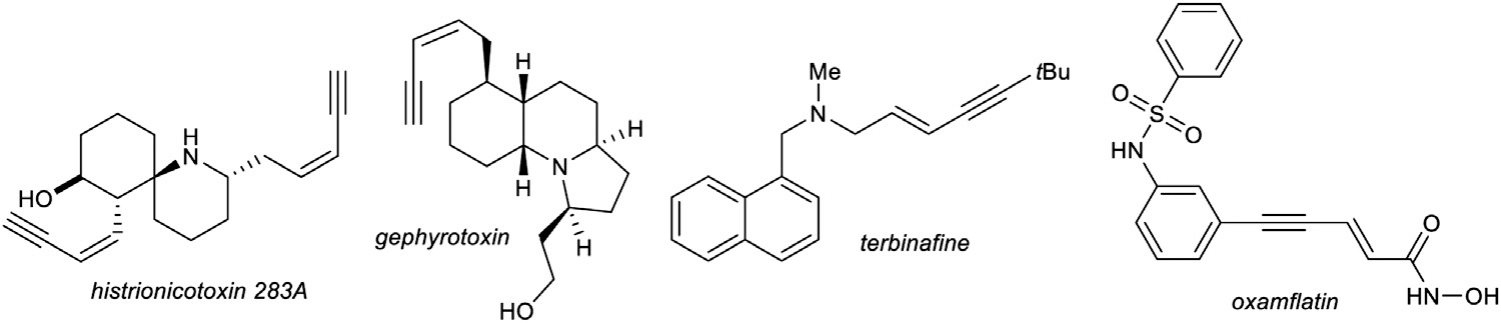
Selected examples of naturally occurring 1,3-enynes and synthetic drugs, which incorporate an 1,3-enyne motif.

1,3-Enynes are powerful building blocks in organic synthesis, due to their unique reactivity of the triple bond and/or double bond which can be addressed selectively (Sasaki et al., 2011; Zhou and Moberg, 2013) or react together as one functional group, for example, in cycloaddition reactions (Gevorgyan and Yamamoto, 1999; Pünner and Hilt, 2012; Röse et al., 2015). The structure motif of 1,3-enynes can be found in several natural products, e.g., histrionicotoxins (*histrionicotoxin 283A*) (Stork and Zhao, 1990) and gephyrotoxin (Daly et al., 1977), found in the skin of poison frogs of the family *Dendrobates histrionicus*, in drugs, e.g., terbinafine, which acts as an antifungal (Leyden, 1998) or oxamflatin, which acts as a HDAC inhibitor (Kim et al., 1999), and moreover in functional materials (Figure 1). (García-Garrido, 2014).

**Figure:**
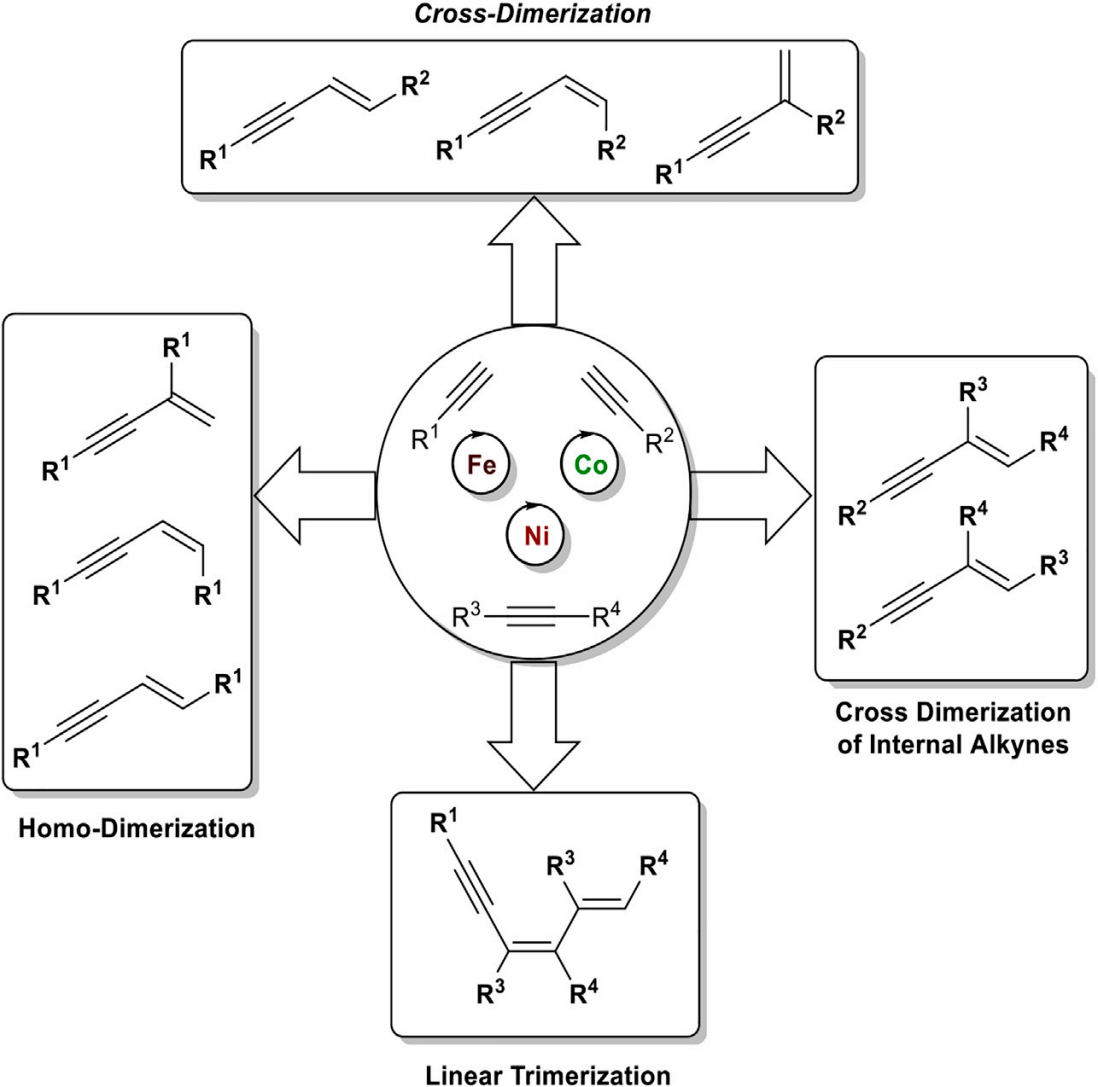


In recent years, several synthetic routes toward 1,3-enynes have been described. One of the most used synthetic methods is palladium-catalyzed cross-coupling reactions, e.g., Sonogashira reactions (Adamson et al., 2019) or Heck-type reactions (Wen et al., 2011) which are generally performed in excellent yields and good regio- and defined stereoselectivities (García-Garrido, 2014). Besides, the Wittig olefination (Karatholuvhu and Fuchs, 2004) of propargylic aldehydes/ketones, elimination reactions starting from propargylic alcohols (Sartori et al., 1996; Ye et al., 2018) for the construction of the carbon-carbon double bond, or rearrangement reactions in Corey-Fuchs-type reactions (Shi Shun and Tykwinski, 2003) of vinyl ketones have been described. One big drawback of all mentioned synthetic methods is the rather low atom economy for olefination or rearrangement reactions and the synthesis of the used starting materials, e.g., alkynyl-bromides or iodides (light-sensitive materials) or vinyl-halides. Because of the sometimes challenging syntheses, the hydroalkynylation (= dimerization) of an easily accessible terminal alkyne with another alkyne is an interesting and highly atom economic approach toward 1,3-enynes (García-Garrido, 2014; Trost and Masters, 2016). Also, some reports with main group (Dash and Eisen, 2000; Hasenbeck et al., 2019) and lanthanide catalysts (Nishiura et al., 2003; Ge et al., 2007; Platel and Schafer, 2012), early transition metal- (Cembellín et al., 2020), but mainly noble-metal-catalyzed dimerization protocols have been published (e.g., Ru (Barsu et al., 2020), Rh (Katagiri et al., 2008), Ir (Ohmura et al., 2000), Pd (Chen et al., 2013)) (Trost and Masters, 2016). Because of their higher toxicity and lower availability, earth-abundant transition metal catalysts, especially iron (Plietker, 2008) and cobalt (Hapke and Hilt, 2020) catalysts, have gained increasing attention in the last years with quite remarkable results (Liang et al., 2020b), which shall be summarized in this review.

**SCHEME 1:**
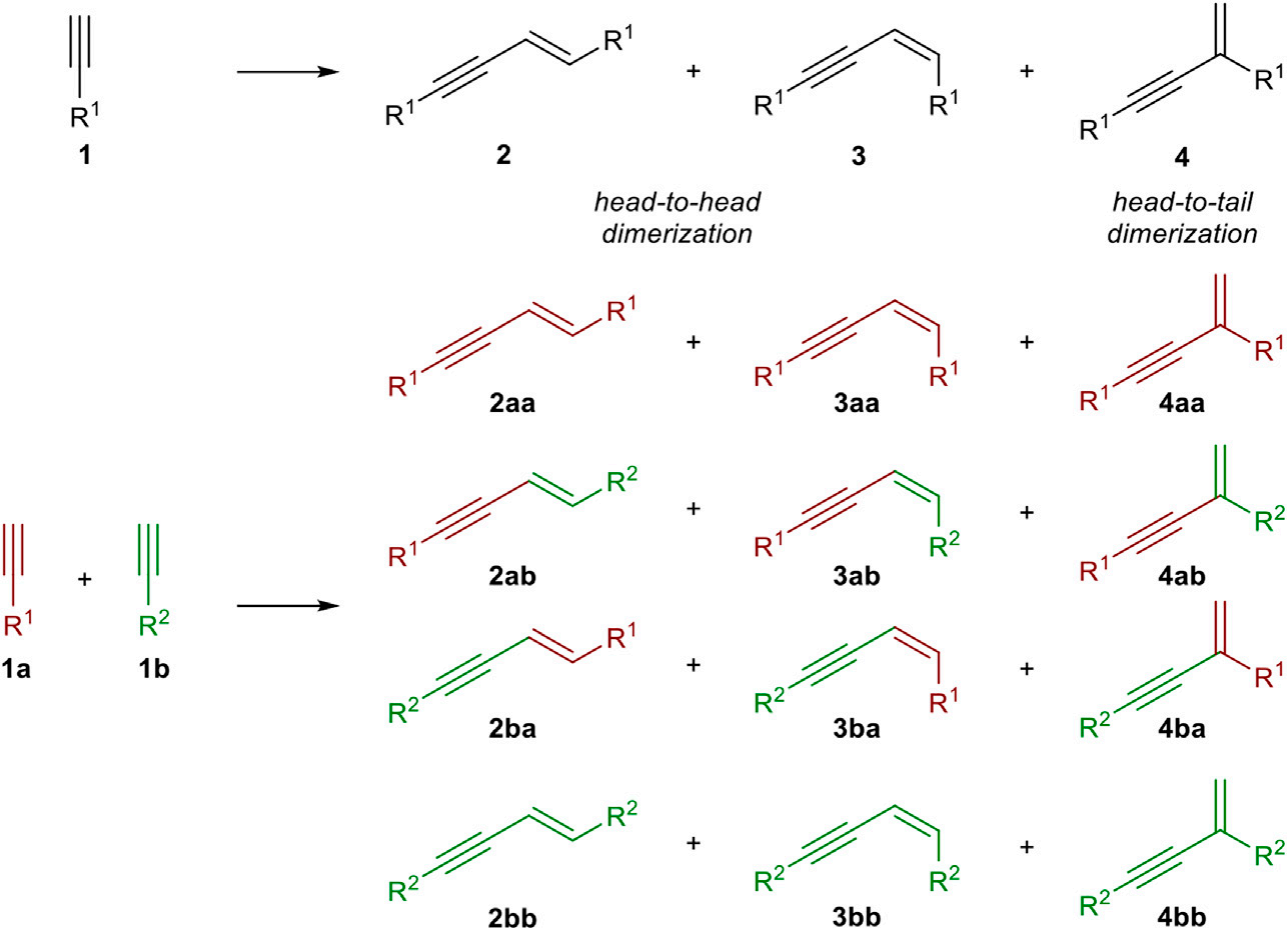
Possible products of the dimerization of terminal alkynes.

**SCHEME 2:**
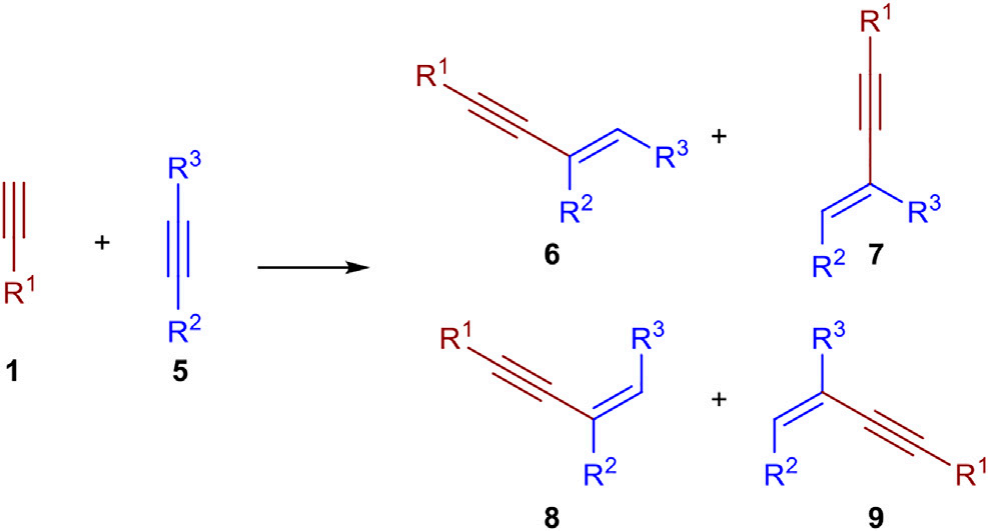
Possible products of the hydroalkynylation of internal alkynes.

The dimerization of two identical terminal alkynes **(1)** leads to three different products. The *head-to-head* dimerization yields internal 1,4-substituted 1,3-enynes with an *E*- **(2)** or *Z*-configured double bond (**3**). The *head*-*to*-*tail* dimerization leads to *geminal* 1,3-enynes (**4**). Also, the formation of cumulenes and oligomerization can occur (García-Garrido, 2014). When two different terminal alkynes are applied to the dimerization process, one donor-alkyne (donates a hydrogen to the other triple bond; its triple bond remains intact) and one acceptor-alkyne (accepts the hydrogen atom, the triple bond reacts to a double bond) are needed. All combinations of homodimerization and cross-dimerization result in 12 different regio- and stereoisomers, excluding oligomerization products (Scheme 1). The hydroalkynylation of internal unsymmetrical alkynes (**5**) with donor-alkynes (**1**) can lead to up to four products (**6–9**) (Scheme 2).

Due to the high number of possible isomers in the dimerization/hydroalkynylation process, the used catalyst must 1) control the chemo-, regio-, and stereoselectivity and 2) suppress further transformation of the 1,3-enynes with additional alkynes toward oligomerization products.

## 2 Iron-Catalyzed Hydroalkynylation

### *E*-Selective *Head-to-Head* Dimerization

**SCHEME 3:**
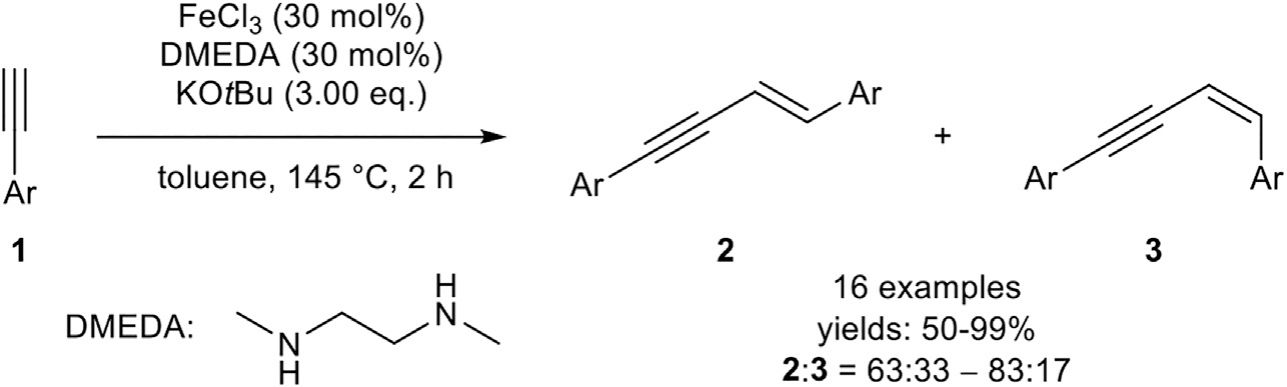
Iron(III)-catalyzed *E*-selective *head-to-head* dimerization (Midya et al., 2011).

For the iron-catalyzed *E*-selective *head-to-head* dimerization, several procedures have been reported including different oxidation states of the metal center (+III, +II, 0). A quite simple catalyst was realized by Dash, consisting of iron(III) chloride (30 mol%), 1,2-dimethylethylenediamine (DMEDA, 30 mol%) as the ligand, and potassium *tert*-butyloxide (3.00 equiv.) in toluene at 145°C (Scheme 3) in a sealed tube (Midya et al., 2011).

The authors could show that all components of the catalyst system are essential for the outcome of the dimerization. In the absence of the ligand, the yield was decreased and the products **2** and **3** were observed in a 1:1 ratio. In the absence of an iron salt, the base KO*t*Bu also promoted the dimerization process, although low conversions and low yields were observed. Moreover, other iron salts, e.g., Fe(acac)_3_, led to low conversions. While a decreasing purity of FeCl_3_ did not affect the dimerization process concerning the yield or the selectivity, anhydrous iron salts were more effective as catalysts. The substrate scope included aryl-substituted terminal alkynes with different electron-donating, electron-neutral, and electron-withdrawing groups, as well as different substitution patterns. While the electronic effects of the substrate did not have a substantial impact on the yield, *meta*-substituted aryl alkynes needed longer reaction times and afforded lower yields than *ortho-*substituted substrates. Surprisingly, alkyl-substituted alkynes did not undergo the dimerization reaction (Midya et al., 2011). Later, the same authors described a similar catalyst system, where the ligand DMEDA was substituted by 1,2-bis(diphenylphosphino)ethane (dppe), which gave marginally better yields. Radical clock experiments indicated that a stepwise radical mechanism, initiated by KO*t*Bu, is present. Unfortunately, alkyl-substituted alkynes were still not tolerated with this catalyst system (Midya et al., 2014).

**SCHEME 4:**
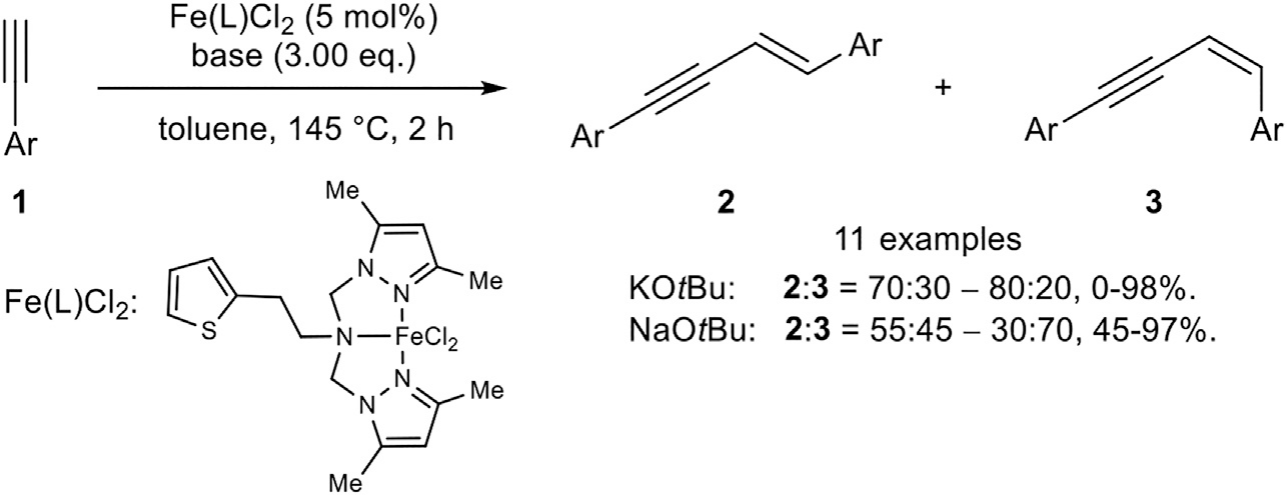
Iron(II)-catalyzed *head-to-head* dimerization (Xue et al., 2018).

Later, Huang reported an iron(II) catalyst system with a tripodal pincer-type ligand where the stereoselectivity is controlled by the cation of the base (Scheme 4) (Xue et al., 2018).

The use of KO*t*Bu as base (3.00 equiv.) led predominantly to the *E*-isomer **2** in up to 98% yield (ratio **2**:**3** = 70:30 up to 80:20). By switching the cation from potassium to sodium the stereoselectivity of the *head-to-head* dimer was inverted to the *Z*-isomer **3** (**2**:**3** = 55:45–30:70) with yields ranging between 45 and 97%. The substrate scope showed that electron-donating substrates gave the highest yields. Moreover, microwave irradiation decreased the reaction time and gave higher yields for the *E*-isomers with comparable selectivities. In contrast, the *Z*-selective reaction with NaO*t*Bu did not proceed under microwave irradiation. Radical clock experiments did slightly diminish the yield, so that the authors excluded a radical mechanism for the iron(II)-catalyzed dimerization (Xue et al., 2018).

**SCHEME 5:**
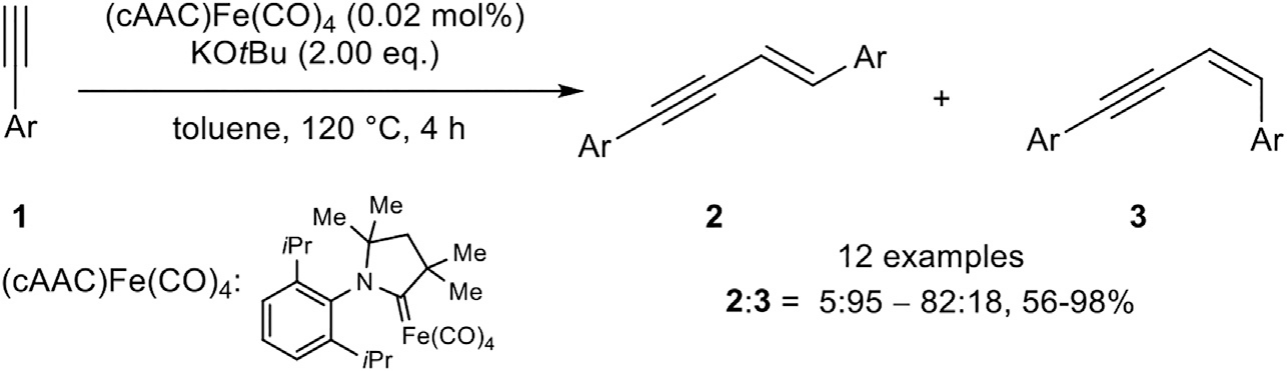
*E*-Selective iron(0)-catalyzed dimerization of terminal alkynes (Bhunia et al., 2016).

Also, Mandal utilized an iron(0) complex which catalyzed the *head-to-head* dimerization with a very low catalyst loading (0.2 mol%) and KO*t*Bu as base in toluene at 120°C (Scheme 5) (Bhunia et al., 2016).

The stereoselectivity was predominantly *E*-selective (**2**:**3** = 5:95–82:18), although 3,5-bis(trifluoro-methyl)phenyl acetylene and 2-ethynylpyridine inverted the selectivity toward the *Z*-isomer. All in all, good to excellent yields between 56 and 98% could be achieved. The substitution pattern showed the highest impact on the yield, where *ortho*-substituted aryl alkynes gave the lowest yield, while *meta*-substituted aryl moieties gave similar yields compared to *para*-substituted substrates. Excluding the abovementioned substrates, which led predominantly to the *Z*-isomer, electron-donating and electron-withdrawing groups gave similar yields. Also, the substrate scope did not involve alkyl-substituted alkynes as well.

### *Z*-Selective *Head-to-Head* Dimerization

**SCHEME 6:**
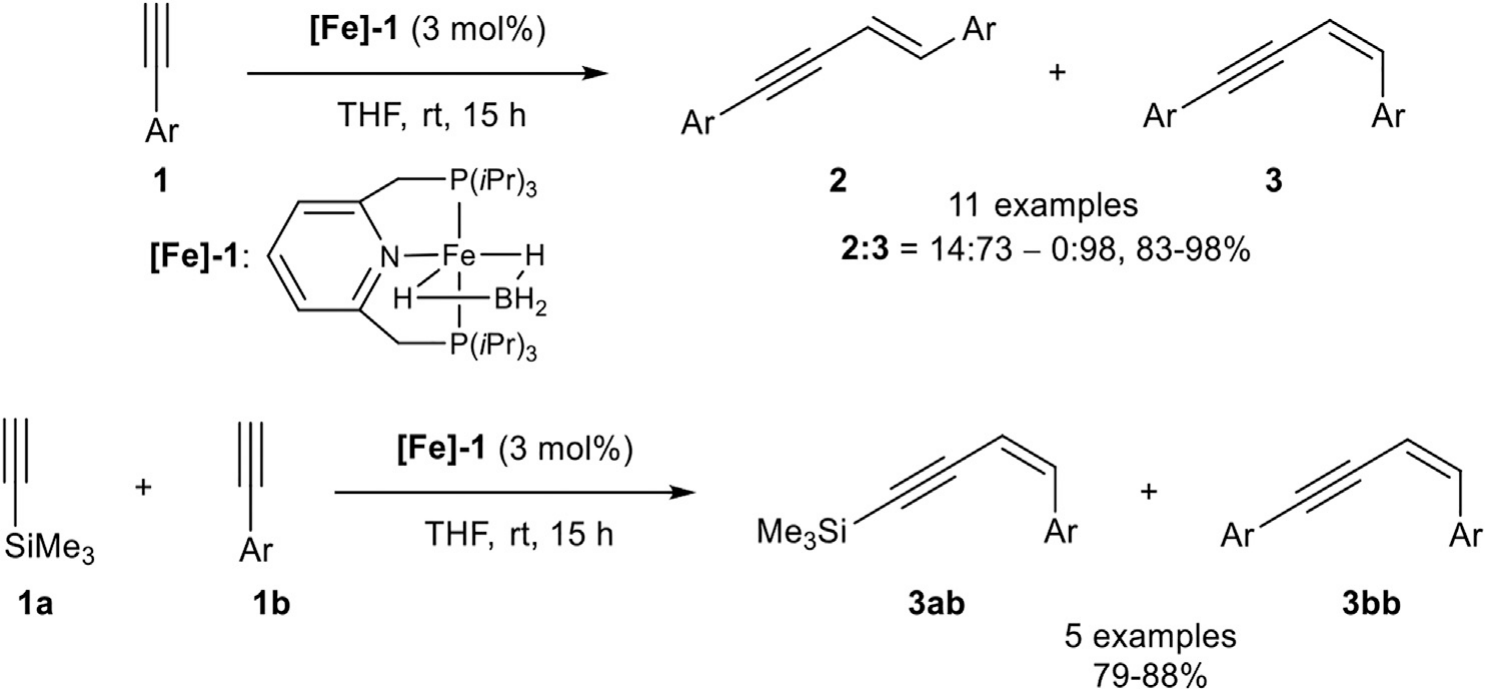
*Z*-Selective dimerization of terminal alkynes (Rivada-Wheelaghan et al., 2016).

In 2016 Milstein reported a *Z*-selective dimerization of terminal alkynes with the PNP-pincer ligand containing iron(II) catalyst **[Fe]-1**. In contrast to the above-described catalyst systems, this catalyst was active in the coordinating solvent tetrahydrofuran without any additives at room temperature. Besides, it was highly *Z*-selective with ratios of **2**:**3** between 14:73 and 0:98 with good to excellent yields (83–98%; Scheme 6).

**SCHEME 7:**
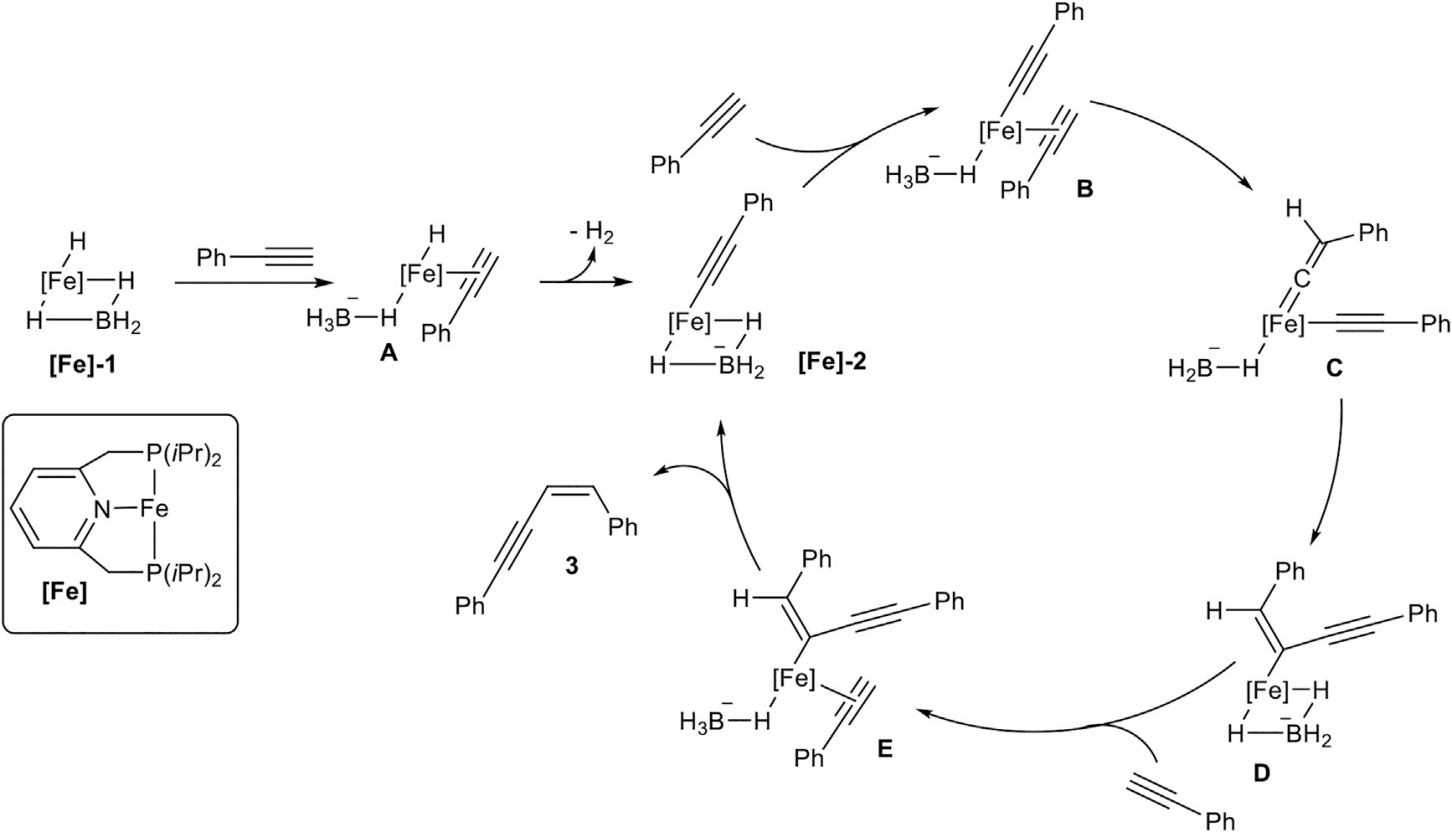
Postulated mechanism for the *Z*-selective dimerization of terminal alkynes catalyzed by [Fe]-1 (Rivada-Wheelaghan et al., 2016).

The substrate scope covered *para-* and *meta*-substituted aryl alkynes with different electron-donating and electron-withdrawing groups. In general, the acidity of the terminal alkyne was decisive for the reaction rate so that substrates with electron-withdrawing groups reacted faster than electron-donating substituents. Unfortunately, the catalyst did not tolerate substrates bearing, neither an *ortho*-substituted aryl alkyne, nor alkyl-substituted substrates. Also, a cross-coupling reaction between trimethylsilyl acetylene (3.00 equiv.) as donor-alkyne and aryl alkynes (1.00 equiv.) to form the *Z-*configured products of type **3ab** in 79–88% yield for five examples was realized. Surprisingly, if trimethylsilyl acetylene was subjected to the dimerization process, only the *gem*-product **4** was generated in 80% yield after 36 h reaction time. Mechanistic insights could be achieved by NMR-spectroscopy and X-ray crystallography of precipitated complexes, generated by the reaction of complex **[Fe]-1** with an excess phenyl acetylene. The authors postulated the following mechanism, which is illustrated in Scheme 7 (Rivada-Wheelaghan et al., 2016).

At first, the catalyst **[Fe]-1** coordinates one alkyne to form complex **A** with an acidified proton at the alkyne, followed by elimination of dihydrogen, consisting of Fe-H and the proton of the alkyne. This suggestion is substantiated by the observation that internal alkynes do not react with the catalyst system. The formed complex **[Fe]-2** was confirmed by X-ray spectroscopy. Starting from **[Fe]-2** another alkyne coordinates to the metal center (**B**), followed by a transformation of **B** into the vinylidene complex **C**. The intramolecular coupling of a carbon-atom from vinylidene fragment with the alkynyl ligand leads to the *Z*-configured double bond in complex **D**. Coordination of another alkyne leads to the formation of complex **E**. This complex undergoes a proton transfer from the coordinated alkyne to the enyne-fragment resulting in the release of product **3** and regeneration of the active species [**Fe]-2**.

**SCHEME 8:**
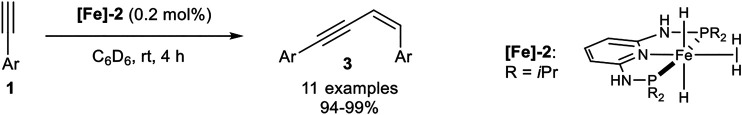
Iron-catalyzed *Z*-selective dimerization of terminal alkynes catalyzed by an PNP-iron(II) complex (Gorgas et al., 2017).

In 2017 Kirchner reported a *Z*-selective dimerization protocol with PNP-pincer-iron(II) complexes with higher catalyst reactivity so that a decreased catalyst loading (0.2 mol%) could be applied. The used PNP-ligand is quite similar to the above illustrated PNP-ligand utilized by Milstein, except for the CH_2_-bridge between the pyridine ring and the phosphine which had been substituted by NH-bridges. Also, the boron tetrahydride ligand was exchanged by two hydride ligands and dihydrogen, bounded by agostic interactions to the iron center. This complex was easily synthesized by the reaction of iron(II) bromide with the PNP-ligand, followed by the addition of 2.00 equivalents of sodium aluminum hydride and hydrolysis with 6.00 equivalents of water. The substrate scope consisted of terminal aryl-substituted alkynes, which generated the dimerization product **3** in excellent isolated yields and excellent stereoselectivities (Scheme 8) (Gorgas et al., 2017).

After that, Kirchner investigated the mechanism by X-ray structure analysis of isolated complexes, trapping experiments with phosphine ligands. Parallel performed DFT calculations supported the mechanistic proposal (Gorgas et al., 2018).

### *Head-to-Tail* Dimerization

**SCHEME 9:**
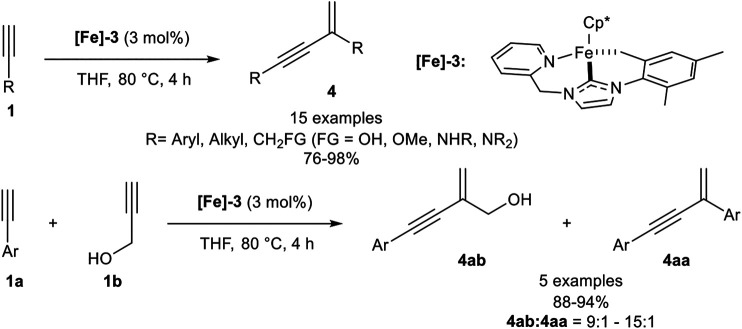
Iron-catalyzed *head-to-tail* dimerization (Liang et al., 2017).

In 2017 Song reported a *gem*-specific dimerization protocol using a piano-stool type iron(II) catalyst **[Fe]-3**. The ligand sphere of this catalyst includes a pentamethylcyclopentadienyl ligand (Cp*) and a tridentate NHC ligand. The active catalyst was synthesized in two steps via ligand exchange reaction from Cp*Fe(TMEDA) with the *in situ* generated NHC ligand, followed by deprotonation of one methyl-group of the mesitylene residue utilizing LiHMDS as base. The catalyst converted terminal alkynes to the *gem*-enyne **4** without any additives at 80°C in THF selectively (Scheme 9) (Liang et al., 2017).

The investigated substrate scope revealed that electron-donating and electron-neutral aryl moieties with *para-* and *meta-*substitution patterns worked well giving high yields (>95%) and high stereoselectivities while *ortho*-substituted aryl substituents such as mesitylene, due to its high steric hindrance, and electron-withdrawing groups, such as ester-substituents, were not tolerated. In contrast to all other dimerization processes, this *head-to-tail* dimerization also proceeded well with alkyl-substituted terminal alkynes. Additionally, primary and secondary alcohols as well as primary, secondary, and tertiary amines were tolerated in good to excellent yields. The dimerization of trimethylsilyl acetylene gave the desired product **4** in 86% yield without traces of the *head-to-head* dimerization products. After these remarkable results, Song investigated the cross-dimerization of aryl-substituted alkynes with a slight excess of a propargylic alcohol (1.30 equiv.). The investigation revealed that the cross-dimerization product can be obtained with excellent yields ranging from 88 to 94%, accompanied with the homodimerization product of the aryl alkyne in good regioisomer ratios between 15:1 and 9:1 (Liang et al., 2017).

**SCHEME 10:**
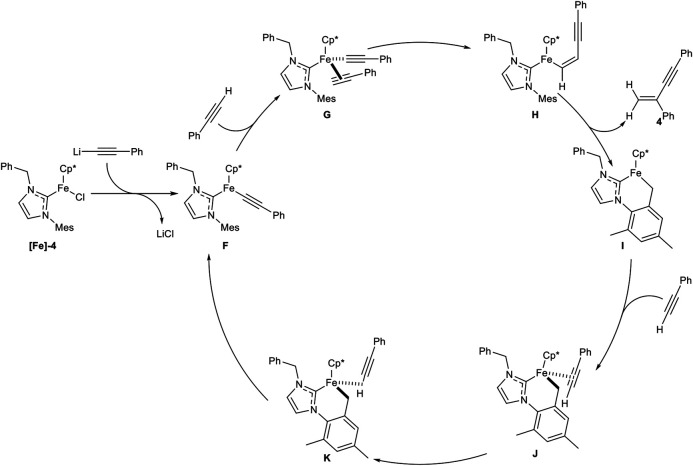
Postulated mechanism for the iron-catalyzed *gem*-specific dimerization of terminal alkynes (Liang et al., 2019).

A second generation of piano-stool-type iron complexes was reported, where the pyridine residue of the NHC ligand was substituted by different alkyl and benzyl substituents. The highest catalytic activity was found with the NHC ligand bearing a benzyl substituent (**[Fe]-4**). In contrast to the above shown first generation piano-stool iron(II) complex **[Fe]-3**, the reaction temperature could be decreased to room temperature and the precatalyst **[Fe]-4** was easily activated by the equimolar addition of LiHMDS to the reaction mixture. The substrate scope showed similar yields and selectivities concerning different aryl- and alkyl-substituted alkynes with similar functional group tolerance. While an ester moiety was accepted in the dimerization step (20% yield), *ortho*-substituted alkynes were still not compatible, due to their high steric demand. Unfortunately, primary alcohols were not tolerated anymore. In the next step, cross-dimerization between *N,N*-dimethyl propargylamine as donor-alkyne (2.00 equiv.) and aryl-substituted alkynes was achieved in good to excellent yields and the following reaction mechanism was proposed (Scheme 10) (Liang et al., 2019).

At first the precatalyst **[Fe]-4** is activated by lithium alkynylide, generated by deprotonation of an alkyne via LiHMDS, to form complex **F**. Coordination of another alkyne leads to complex **G**, followed by migratory insertion to give complex **H**. σ-Bond metathesis releases the *gem*-enyne product **4** and the cyclometalated complex **I.** This complex is coordinated by another alkyne to form the η^2^-coordinated complex **J**, which isomerizes in the rate-determining step into the complex **K**. This complex undergoes another σ-bond metathesis into the active species **F** (Liang et al., 2019; Liang et al., 2020a). The high regioselectivity in this catalysis can be explained by complex **G**. If the alkyne would coordinate in the inverted manner, the *head-to-head* dimerization product would be released. Because of the high steric hindrance of the mesitylene residue, the alkyne can only coordinate in a facile fashion, so that the *gem*-enyne product is highly preferred.

## 3 Cobalt-Catalyzed Hydroalkynylation

Cobalt catalysts used in organic synthesis involve different oxidation states (0,+I,+II,+III) of the active species (Gläsel and Hapke, 2020). Typical transformations including alkynes are cycloadditions reactions, e.g., Diels-Alder reactions (Hilt et al., 2006) or cyclotrimerization reactions (Vollhardt, 1984), but also Alder-ene-type reactions (Hilt and Treutwein, 2007), whereas the oxidation state of the active species ranges from 0 to + I. Besides, cobalt is also used in C-H activation reactions with either cobalt(0) or cobalt (I) complexes (Halbritter et al., 1994; Gao et al., 2010; Fallon et al., 2015; Moselage et al., 2015) or cobalt(III) complexes as the active species (Yoshino et al., 2013; Zhao et al., 2015; Moselage et al., 2016).

### *E*-Selective *Head-to-Head* Dimerization

The chemo- and regioselective dimerization of terminal alkynes with cobalt catalysts had been quite difficult, due to the predominant cyclotrimerization toward trisubstituted benzene derivatives. Our group had observed that *in situ* generated cobalt(I) phosphine catalysts, reduced by magnesium, lead to the formation of the *E*-configured *head-to-head* dimerization product and the cyclotrimerization product (Hilt et al., 2005a).

**SCHEME 11:**
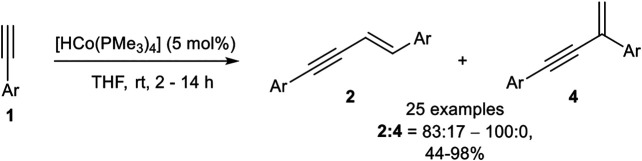
Cobalt(I)-catalyzed *E*-selective dimerization of two alkynes (Ventre et al., 2013).

In 2013 Amatore, Aubert, Petit, and coworkers reported an *E*-selective dimerization protocol with a hydrido-cobalt phosphine complex as catalyst. Different cobalt(I) phosphine complexes were tested, where the anionic ligand was substituted by a chloro or a methyl ligand, which gave inferior results. Moreover, the *in situ* generation of the hydrido-complex decreased the yield of the dimerization product. The optimized reaction conditions are depicted in Scheme 11 (Ventre et al., 2013).

**SCHEME 12:**
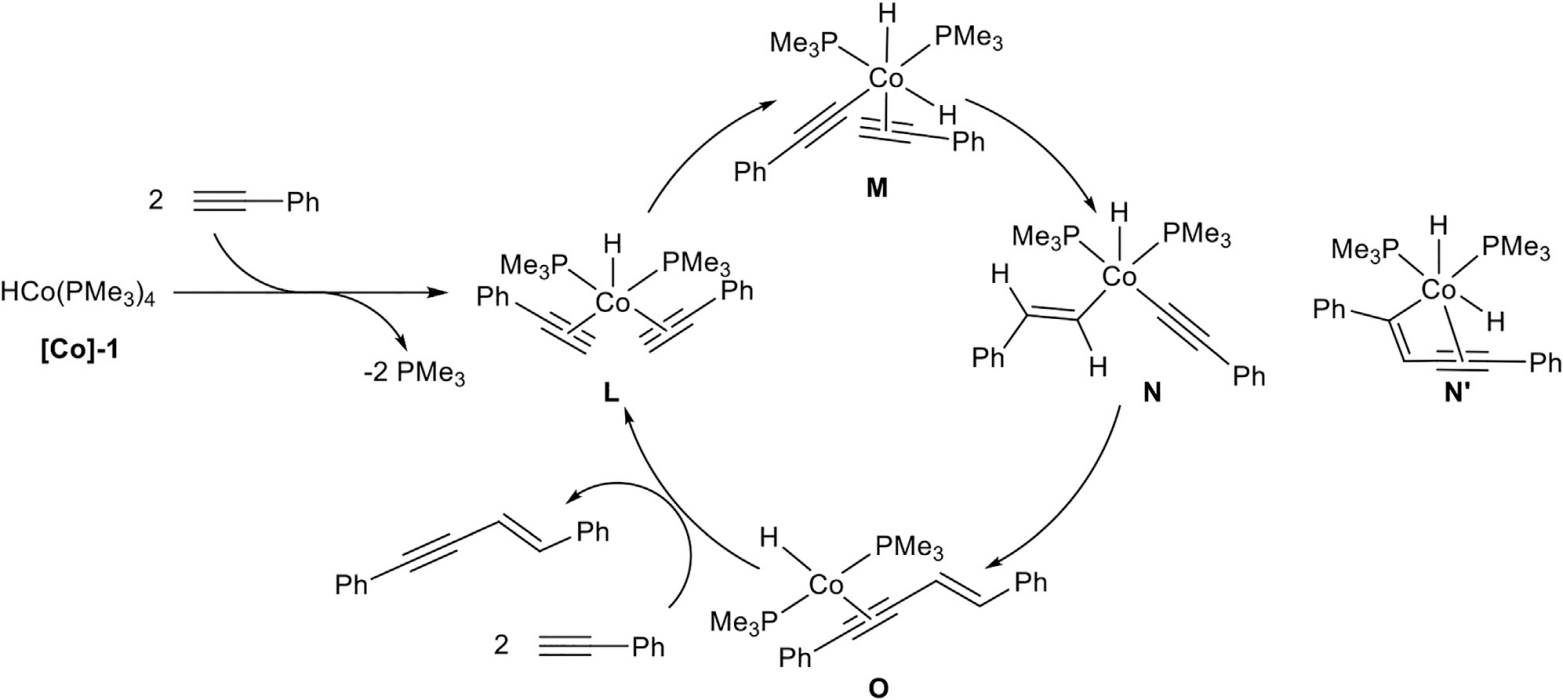
Postulated mechanism of the cobalt-catalyzed *E*-selective *head-to-head* dimerization toward product 2 (Ventre et al., 2013).

The investigation of the substrate scope covered aryl alkynes with *ortho*-*, meta*-, and *para-*substituted residues with electron-donating, electron-neutral, and electron-withdrawing groups. Halogen atoms were also tolerated, excluding iodide. Heteroaromatic compounds such as 3-pyridine or 2-thiophene could be dimerized in good yields. 2-Pyridine did not give a full conversion of the starting material, possibly due to the coordination ability of the pyridine ring toward the central atom. Also, aliphatic alkynes led to a complex mixture of products, consisting of the dimerization product and higher oligomers. All in all, 25 examples have been reported with yields between 44 and 98% in good to excellent regioselectivities between the *E*-configured *head-to-head* product and the branched product. The regioselectivity between the branched product and the *head-to-head* dimer **2** depended on the substitution pattern of the aryl-moiety. While *para*-substituted aryl moieties gave exclusively the product **2**, *meta*- and *ortho*-substituted substrates with methyl or methoxy residues led to a decrease in the selectivity giving regioisomeric ratios of 83:17, still favoring the *head-to-head* dimer **2**. The cross-dimerization of two different substituted alkynes was also investigated, but only 1-hexyne and silyl ethers led to moderate yields of the cross-product. Based on DFT calculations the following mechanism (Scheme 12) had been postulated (Ventre et al., 2013).

A ligand exchange of **[Co]-1** initiated by two terminal alkynes leads to complex **L** by the release of two phosphine ligands. An oxidative C-H activation of one alkyne ligand results in complex **M**, bearing two hydrido ligands with cobalt(III) as the central atom. Then a hydrocobaltation takes place and complex **N** is formed, followed by a reductive elimination toward complex **O**. The product is released via ligand exchange, initiated by two alkynes to regenerate the complex **L** and closing the catalytic cycle. DFT calculations revealed that a hydrocobaltation is favorable in comparison to a carbocobaltation pathway (complex **N’**) which would lead to the direct formation of the new carbon-carbon bond (Scheme 12) (Ventre et al., 2013).

**SCHEME 13:**
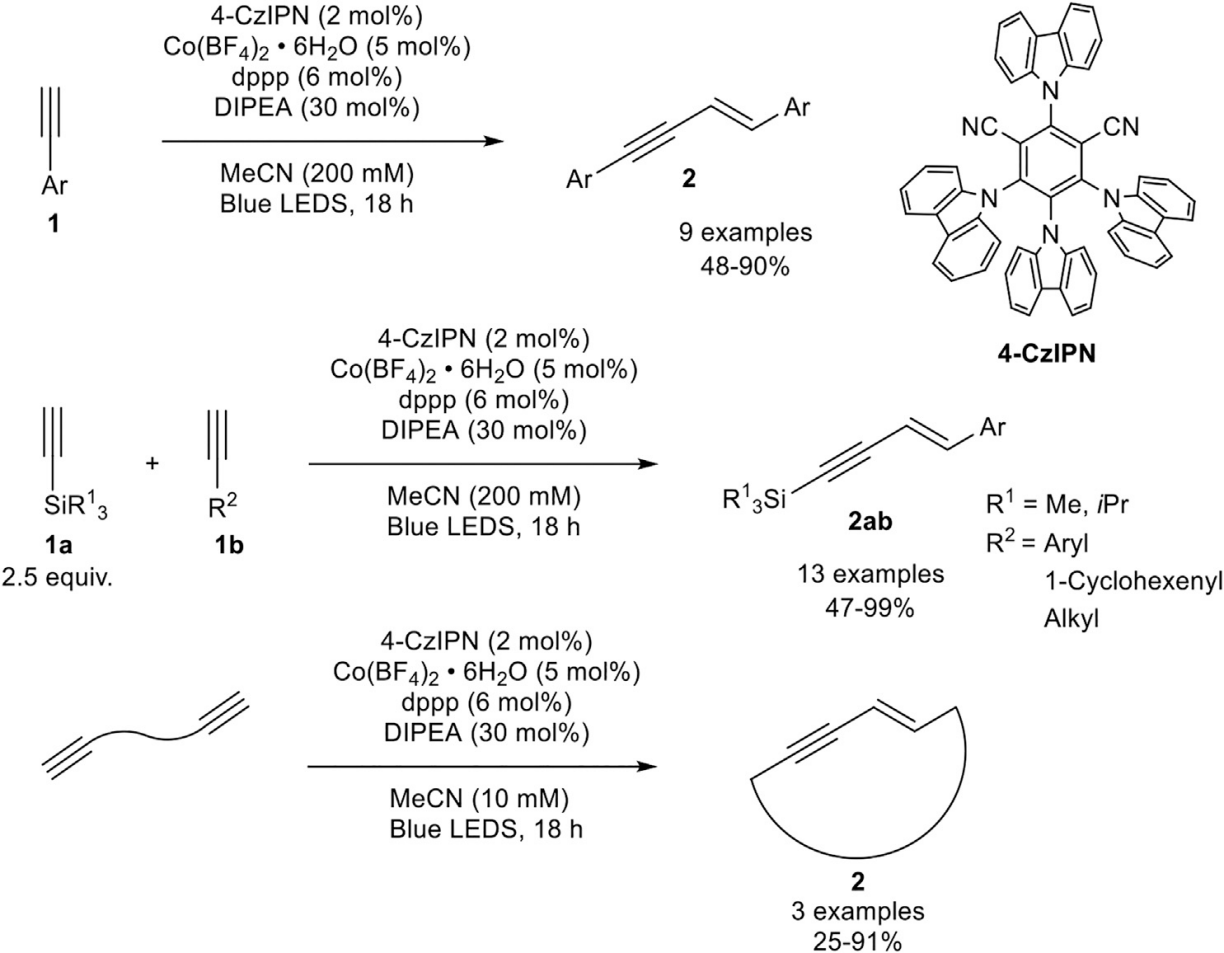
Cobalt-catalyzed (cross-) hydroalkynylation of terminal alkynes and α,ω-diynes (Grenier-Petel and Collins, 2019).

An interesting alternative catalyst system had been developed by Collins, who activated the precatalyst by photoredox catalysis. The catalyst system consists of cobalt(II) tetrafluoroborate, 1,3-bis(diphenylphosphino)propane as the bidentate ligand, di-*iso*-propylethylamine, and the photocatalyst 4-CzIPN (see Scheme 13) in acetonitrile, which is irradiated with blue light emitting LEDs. During their optimization studies the authors observed that the dimerization reaction proceeds only in the presence of irradiation. Also, other ligands and other cobalt salts resulted in lower yields or the catalyst system led to the formation of the cyclotrimerization product. The optimized reaction conditions are illustrated in Scheme 13 (Grenier-Petel and Collins, 2019).

With the optimized reaction conditions in hand, the substrate scope of the homodimerization was investigated. Electron-donating groups (e.g., methoxy, methyl) and halogen atoms, excluding iodide, as well as protected aniline derivatives were well tolerated. Additionally, 6-methoxynaphtyl and 1-cyclohexenyl acetylene could be homodimerized in good yields. In the next step, the authors investigated the cross-dimerization between two different alkynes. The substrate scope revealed that trialkylsilyl acetylenes could be used as donor-alkynes in the hydroalkynylation to afford the cross-product **2ab** in moderate to excellent yields (47–99%). The yield depended on the used equivalents of the silyl-substituted alkyne: While 1.50 equivalents of the donor-alkyne led only to 23% yield, the increase to 2.50 equivalents led to 91% yield in their test system. A further increase of the equivalents did not increase the yield significantly. Furthermore, α,ω-diynes could be homodimerized toward large 17-19-membered ring 1,3-enynes by decreasing the substrate concentration. In contrast to the developed catalyst by Petit, the authors postulated the reduction of the precatalyst to cobalt(0) by irradiation so that a Co(0)/Co(II) catalytic cycle is present (Grenier-Petel and Collins, 2019).

**SCHEME 14:**
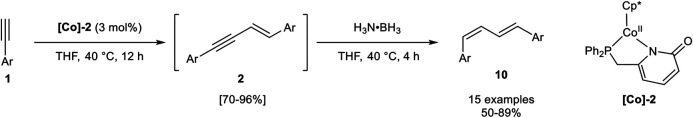
Cobalt-catalyzed sequential one-pot synthesis of *E*,*Z*-configured 1,3-dienes 10 via *E*-selective dimerization and chemoselective semihydrogenation (Zhuang et al., 2019).

In 2019 Liao and Wang reported a sequential one-pot procedure for the generation of 1,4-substituted *E*,*Z*-configured 1,3-dienes **10** via an *E*-selective hydroalkynylation semihydrogenation sequence. The investigated Co(II) complex **[Co]-2** was able to stereoselectively dimerize aryl-substituted terminal alkynes of type **1** into the intermediate **2**. Only 4-trifluoromethylphenyl acetylene gave lower stereoselectivities, where the *Z*-configured product **3** was detected (stereoisomeric ratio **2**:**3** = 86:11). The 1,3-enyne intermediate **2** could be isolated after purification in good to excellent yields or the 1,3-dienes could be generated by the addition of two equivalents of borazane (H_3_N·BH_3_) to the reaction mixture (Scheme 14) (Zhuang et al., 2019).

The investigated substrate scope consisted of aryl-substituted alkynes with different substitution patterns and electron-donating, electron-neutral, and electron-withdrawing groups. All tested substrates gave moderate to excellent yields of 50–89% in this one-pot reaction sequence. Notably, chloro-substituted substrates gave the lowest yields. While alkyl-substituted alkynes have not been described, trimethylsilyl acetylene could be transformed into the corresponding 1,3-enyne preferring product **2** (**2**:**3** = 57:19). The hydrogenation of this substrate has not been described. Mechanistic investigations based on crystallized cobalt-acetylide complexes and DFT calculations led to a postulated mechanism, similar to the iron(II)-catalyzed mechanism described by Song (Scheme 10) for the dimerization process. The hydrogenation toward product **10** should be realized by coordination of the ammonia-borane complex toward the cobalt center of **[Co]-2** giving a hydrido-cobalt complex as the active species. Interestingly, the DFT calculations revealed that this transformation does not involve oxidative addition and reductive elimination steps so that all oxidation states of the cobalt-atom remain + II in the catalytic cycles (Zhuang et al., 2019).

**SCHEME 15:**
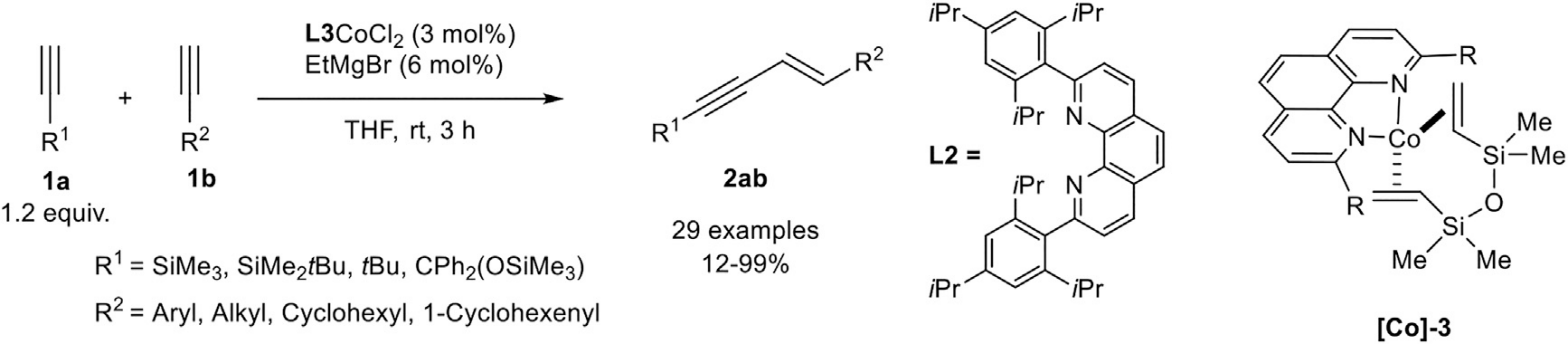
Cobalt-catalyzed *E*-selective cross-dimerization between a bulky donor-alkyne and an acceptor-alkyne (Ueda et al., 2020).

A very effective cross-dimerization of a donor-alkyne and an acceptor-alkyne had been developed by Tsurugi, Mashima, and coworkers, who used a cobalt(II) phenanthroline complex as precatalyst, which was *in situ* reduced by ethyl magnesium bromide in THF. In their investigation, the authors tested different 2,9-disubstituted phenanthroline ligands with a varying steric demand of the substituents. The best results in the cross-dimerization of trimethylsilyl acetylene as the donor-alkyne **1a** and an acceptor-alkyne **1b** were achieved with the bulky phenanthroline derivative **L2** as ligand (Scheme 14). Also, ethyl magnesium bromide turned out to be the best reducing agent, while other reducing agents, e.g., other Grignard reagents, Zn or AlMe_3_, led to inferior results. It is notable that this catalyst gave high yields and high regioselectivities between the cross-dimerization product **2ab** and the homodimers with only 1.20 equivalents of the donor-alkyne **1a**. Additionally, no other enyne derivative (*Z*-enyne **3** or the *gem*-enyne **4**) was observed (Scheme 15) (Ueda et al., 2020).

The substrate scope revealed a high functional group tolerance of the catalyst system. Electron-donating groups (hydroxy, alkoxy), as well as electron-neutral and electron-withdrawing groups (cyano, ester, halogen), were well tolerated. Although aryl and heteroaryl residues are tolerated, the yield of the dimerization product depended on the position of these residues: The highest yields were achieved when a methylene-group is next to the triple bond of the acceptor-alkyne **1b**. The cross-dimerization product of 4-methoxyphenyl acetylene gave 69% isolated yield while 4-trifluoromethylphenyl acetylene gave only 12% yield detected by NMR-spectroscopy. The variation of the donor-alkyne **1a** revealed that bulky substituents, e.g., SiMe_3_, SiMe_2_ *t*Bu, or CPh_2_(OSiMe_3_), gave excellent yields, while 3,3-dimethyl-1-butyne increased the yield to 61%, indicating that the position of the bulky substituent affects the outcome of the reaction. This observation may be explained by the steric interaction of the ligands with the donor-alkyne after the C-H-activation step (compare Scheme 12, compound **M**). At last, the authors crystallized the complex **[Co]-3** by reduction of the precursor and addition of divinyltetramethyldisiloxane. This cobalt(0) complex was subjected to the cross-dimerization process as the catalyst, which gave the desired product in similar yields. Due to this observation the authors postulate a Co(0)/Co(II) catalytic cycle (Ueda et al., 2020), similar to the postulated mechanism by Petit (Scheme 12) (Ventre et al., 2013).

Recently, Thomas reported a heterobimetallic Zr/Co-complex which was able to promote the dimerization of terminal alkynes. The hydroalkynylation of phenyl acetylene with a catalytic amount of this bimetallic complex led to the formation of a polymer, due to the high activity toward the primary formed 1,3-enynes (Beattie et al., 2020).

**SCHEME 16:**
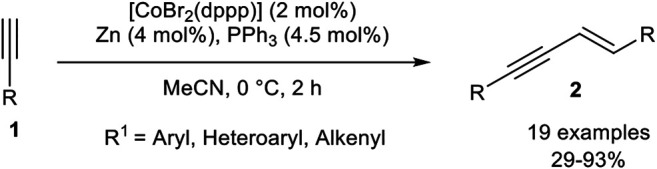
*E*-Selective cobalt(I)-catalyzed homodimerization of terminal alkynes toward *head-to-head* dimers (Weber et al., 2020).

Our group developed a catalyst system consisting of CoBr_2_(dppp), triphenylphosphine, and zinc as reducing agent in acetonitrile. In comparison to our previous reported catalyst systems concerning cyclotrimerization reactions (Hilt et al., 2005b; Weber and Hilt, 2019), the addition of triphenylphosphine and the absence of the Lewis acid led to a complete change in the selectivity toward the *head-to-head* dimer **2** (Scheme 16) (Weber et al., 2020).

The optimization of the reaction conditions was conducted via *Design of Experiments*, which reveals that a low catalyst loading of 2 mol% gave higher yields than a higher catalyst loading of 10 mol%. Also, lower reaction temperatures increased the yield of the desired product **2**. The equivalents of triphenylphosphine were set to 2.25 equivalents with respect to the catalyst loading as the optimized condition. To our surprise, the addition of zinc iodide as a Lewis acid resulted in a decrease of the yield, indicating that the abstraction of the remaining bromide ligand of the active cobalt(I) catalyst is counterproductive. Other solvents, as well as other cobalt sources and ligands, were tested before the optimization of the categorical and continuous parameters. With the optimized conditions in hand, we investigated the substrate scope of the homodimerization: In general aryl acetylenes with electron-donating groups, as well as electron-neutral and electron-withdrawing groups, were well tolerated. Also, halogen-substituents were tolerated well, where the yield showed the trend that fluorine gave lower yields than its higher homologues chlorine and bromine. In general, the best yields were achieved when the product precipitated out of the reaction mixture. Different substitution patterns of the aryl-moiety showed that *para*-substituted aryl residues gave the best results, but surprisingly starting materials with *ortho*-substituents gave higher yields than those with *meta*-substituted aryl alkynes. Heteroaromatic alkynes, such as thienyl or pyridinyl, as residues were tolerated, even 2-ethynylpyridine, which is difficult to dimerize due to the likely coordination of the nitrogen-atom toward the central atom of the catalyst. At last, 2-methyl-but-1-en-3-yne was tolerated and the product was obtained in 29% yield. All tested aliphatic alkynes and trimethylsilyl acetylene gave no conversion or oligomerization of the starting material (Weber et al., 2020). We believe that zinc reduces the precatalyst [CoBr_2_(dppp)] to [CoBr(dppp)], which stabilizes itself by the addition of two triphenylphosphine ligands to form the pentacoordinated complex [CoBr(dppp)(PPh_3_)_2_]. The mechanism should be similar to the calculated mechanism by Petit as outlined in Scheme 12.

### *Z*-Selective *Head-to-Head* Dimerization

**SCHEME 17:**
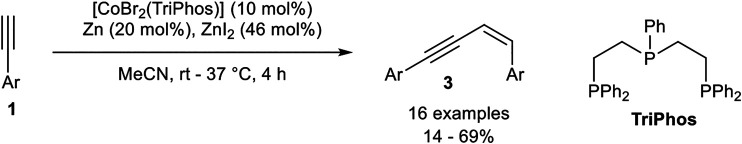
Cobalt(I)-catalyzed *Z*-selective *head-to-head* dimerization (Weber et al., 2020).

Only a few examples of *Z*-selective head-to-dimerization toward enynes of type **3** are reported. One example was developed by our group. Changing from the bidentate ligand dppp to the tridentate linear bis(2-diphenylphosphinoethyl)phenylphosphine (TriPhos) led to an inverted stereoselectivity of the *head-to-head* dimerization. The optimization of the reaction via *Design of Experiments* revealed that an almost equimolar amount of zinc iodide (with respect to the product) is needed. Also, a higher catalyst loading of 10 mol% and a higher temperature between room temperature and 37°C was needed (Scheme 17) (Weber et al., 2020).

The substrate scope included the same functional group tolerance, except alcohols, 2-ethinylpyridine, and vinyl residues. In general, low to moderate yields between 14 and 69% could be achieved with high stereoselectivities toward the *Z*-configured product. *Para*- and *meta-*substituted aryl moieties gave the highest selectivities of up to 99:1, while *ortho*-substituted aryl moieties led to a decreased *E:Z*-selectivity (41:59). Also, aliphatic alkynes were not tolerated and led to no conversion or the formation of oligomers of unknown constitution. The role of zinc iodide in this dimerization process has not been completely understood yet. The change in the selectivity may be explained by using the tridentate linear TriPhos ligand, whereby an altered mechanism is realized (Weber et al., 2020). As the key intermediate in this altered mechanism a vinylidene complex could be reasonable, as described by Milstein (Rivada-Wheelaghan et al., 2016) and Kirchner (Gorgas et al., 2018) before.

**SCHEME 18:**
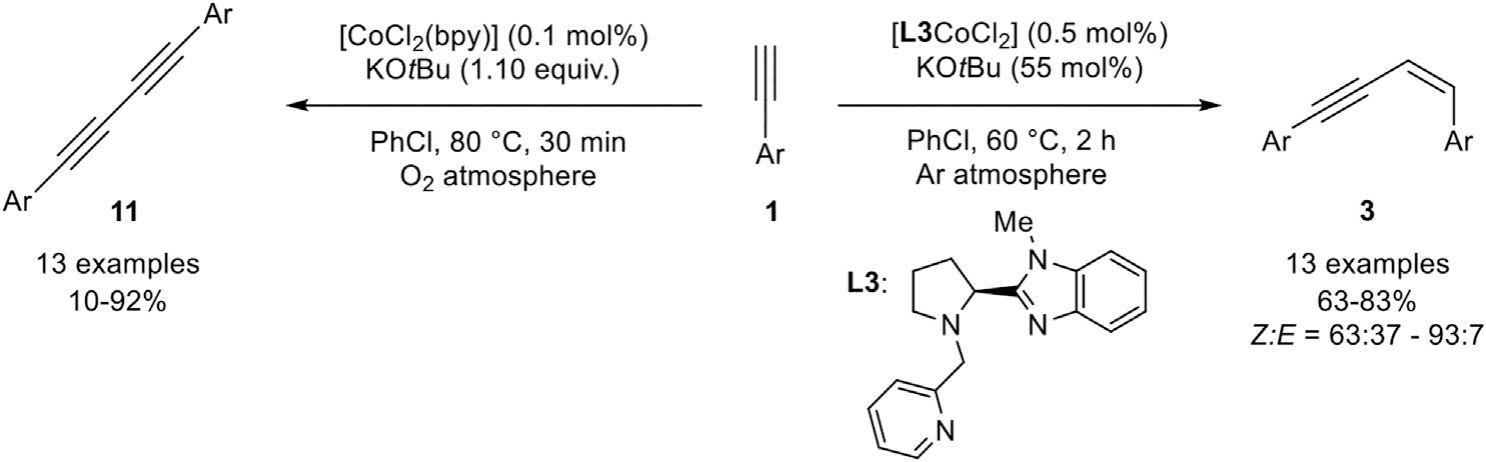
*Z*-Selective homodimerization and Glaser-type coupling of terminal alkynes catalyzed by cobalt complexes (Xu et al., 2018).

Another method to generate *Z*-configured enynes of type **3** was reported by Wang, Sun, and coworkers. The authors reported a cobalt(II)-pincer complex which was able to catalyze the homodimerization of terminal aryl alkynes under argon atmosphere in good to excellent yields with good stereoselectivities toward the *Z*-enyne (Scheme 18) (Xu et al., 2018).

The catalyst [**L3**CoCl_2_] was used in a low catalyst loading of 0.5 mol% with KO*t*Bu as base in a slight excess (with regard to the product) in chlorobenzene at 60°C. The bipyridine derivative [CoCl_2_(bpy)] was also tested as the catalyst and gave similar yields, but a lower *Z*-selectivity. Lower catalyst loadings and lower temperatures led to decreased yields of the product while the stereoselectivities remained unchanged. Different cobalt salts were also investigated, which gave inferior results. Other bases, such as potassium carbonate or triethylamine, gave no conversion of the substrate toward the desired product **3**. The stereoselectivity between the *E-*isomer **2** and the *Z*-isomer **3** depended on the nature of the substrate giving good selectivities preferring the *Z*-isomer (*Z*:*E* = 76:24 –93:7). Only 2-ethinylthiophene gave a diminished selectivity (*Z*:*E* = 63:37). The tested substrate scope included different substitution patterns giving similar results. The electronic nature of the residue did not affect the outcome of the yield. Unfortunately, only aryl residues were tolerated and aliphatic alkynes, such as cyclohexyl acetylene, gave no conversion at all. Interestingly, the catalyst [CoCl_2_(bpy)] catalyzed the oxidative homocoupling of terminal alkynes toward 1,3-diynes **11** with 1.10 equivalents of KO*t*Bu as additive. Different aryl alkynes were subjected to this catalyst system, showing good to excellent yields. Only 4-chlorophenyl acetylene gave a low yield of 10% while 4-bromophenyl acetylene did not give any desired product. The authors explained this observation, by the good leaving group in *para*-position of the substrate leading to an oligomerization of the product. However, chloro-substituents in *meta*- and *ortho*-position gave the product **11** in good yields of 80 and 70% yield. Also, cyclohexyl acetylene could be homocoupled to the 1,3-diyne in 67% yield. First mechanistic investigations with radical clock experiments revealed that no radical mechanism should be present, neither in the dimerization toward product **3** nor in the Glaser-type coupling (Xu et al., 2018).

### *Head-to-Tail* Dimerization

**SCHEME 19:**
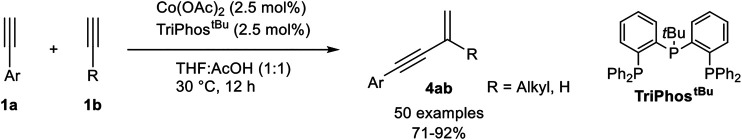
Cobalt(II)-catalyzed *gem*-specific (cross-) dimerization of terminal alkynes (Chen and Li, 2020).

While cobalt-catalyzed cross-dimerization of terminal alkynes normally needs a donor-alkyne with a high steric demand, Li described an Co(II)-TriPhos^tBu^-catalyzed cross-coupling of an aryl-substituted alkyne **1a** and an alkyl-substituted acceptor-alkyne **1b**, which led to the *head-to-tail* dimerization product **4ab** (Scheme 19) (Chen and Li, 2020).

**SCHEME 20:**
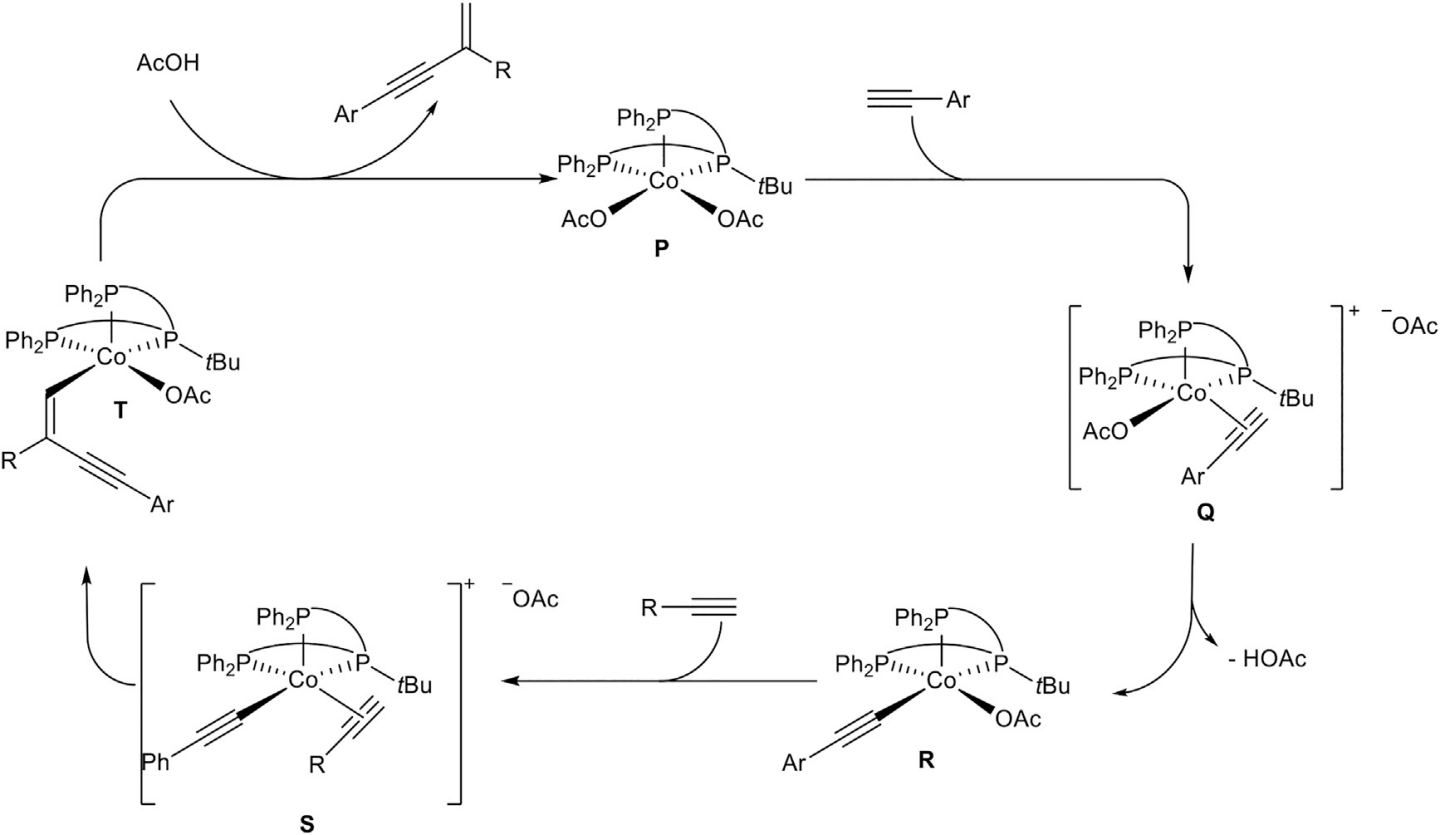
Postulated mechanism of the cobalt(II)-catalyzed *head-to-tail* dimerization of terminal alkynes (Chen and Li, 2020).

The chemoselectivity of this cross-dimerization is determined by the CH-acidity of the used alkynes. The higher acidic alkynes serve as the donor-alkyne **1a** while the other one accepts the hydrogen atom. Different tridentate phosphine ligands were tested and the highest yields were obtained with TriPhos^tBu^ although the linear TriPhos ligand (see Scheme 16) also gave a good yield of 79% and highly preferred the *gem*-product **4ab**. The selectivity and yield depended on the bulkiness of the residue at the central phosphorous atom. Next, different cobalt salts and solvents were tested. It turned out that cobalt acetate gave the best results and that acetic acid as (co)solvent is crucial for the dimerization process. With the optimized reaction conditions different substrates were subjected to the dimerization process. The substrate scope showed that many aryl alkynes underwent the cross-dimerization with alkyl-substituted alkynes in good to excellent yields and excellent chemo- and regioselectivities. All substitution patterns were tolerated and a high functional group tolerance, including electron-donating, electron-neutral, and electron-withdrawing groups, was found. Also, alkenyl-substituted alkynes were able to serve as donor-alkynes in the reaction. Next, the authors varied the acceptor-alkyne **1b**. This variation showed that primary and secondary unsubstituted alkyl moieties, as well as primary, secondary, and tertiary propargylic alcohols, were tolerated as well. Moreover, different functional groups are tolerated at the acceptor-alkyne unit. At last, the authors utilized acetylene gas as the substrate, which turned out to be an effective acceptor-alkyne for the cross-dimerization with aryl-substituted alkynes. Besides cross-dimerization of two different alkynes, the authors also tested the homodimerization of aryl- and aliphatic-substituted alkynes, which resulted in the *gem*-1,3-enyne as the highly preferred product. Based on mechanistic investigations the following mechanism was postulated (Scheme 20) (Chen and Li, 2020).

At first, the more acidic alkyne coordinates to the cobalt complex **P**. After that, the coordinated alkyne is deprotonated by an acetate ligand to give the cobalt-acetylide complex **R**. Ligand substitution by coordination of the acceptor-alkyne to the cobalt center affords the cationic cobalt complex **S**. Due to the steric demand of the *t*Bu-residue of the ligand in direct neighborhood to the coordinated acceptor-alkyne the regioselectivity toward the *gem*-1,3-enyne **4** can be explained. Also, the less hindered acceptor-alkyne with an alkyl substituent coordinates to the metal center in complex **S**, so that cross-dimerization of the reaction is highly preferred. Migratory insertion of the cobalt-carbon bond into the coordinated acetylene gives complex **T**, followed by protonation with acetic acid to release the product **4** and regeneration of the active species **P** (Scheme 20) (Chen and Li, 2020).

### Hydroalkynylation of Internal Alkynes

**SCHEME 21:**
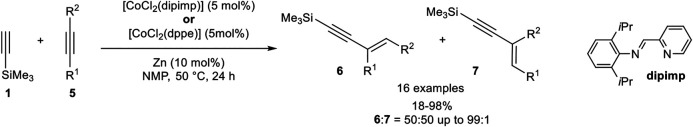
Cobalt(I)-catalyzed hydroalkynylation of internal alkynes (Sakurada et al., 2013).

In contrast to iron catalysis, the cobalt-catalyzed hydroalkynylation of internal alkynes had been demonstrated by Li using CoCl_2_ with a pyridyl-imine or a bidentate phosphine ligand, which was *in situ* reduced to the active cobalt(I) species with zinc as the reducing agent in *N*-methylpyrrolidone (NMP) at 50°C. With (2,6-diisoproyphenyl)-1-(pyridine-2-yl)methanimine as ligand, different diaryl-substituted alkynes **5** were hydroalkynylated by silyl-substituted alkynes **1** in good to excellent yields, using a small excess of 1.30 equivalents of terminal alkyne **1** (Scheme 21) (Sakurada et al., 2013).

When *para*-substituted aryl residues with electron-donating (OMe) as well as electron-withdrawing groups (CO_2_Et) were applied similar results toward the *E*-substituted 1,3-enyne of type **6** were obtained. Interestingly, only activated internal alkynes proceed under the reaction conditions, while unactivated alkynes such as 4-octyne or hept-2-yn-1-ol gave only trace amounts of the desired product. In contrast, 1,4-dimethoxybut-2-yne was converted in almost quantitative yield. Unsymmetrical substituted internal alkynes underwent the hydroalkynylation in moderate yields with only poor selectivities between the regioisomers, in which the former residues of the internal alkynes are *Z*-configured to each other. The use of bidentate phosphine ligands (dppe or dppPh) led to comparable yields and selectivities for diaryl-substituted alkynes **5**. However, unactivated alkynes were still not tolerated and gave no conversion toward the 1,3-enyne product. In case of unsymmetrical substituted activated alkynes, the regioselectivity of the carbon-carbon bond formation could be increased toward the less hindered carbon-atom (**6**:**7** = 60:30 up to >99:1). Also, heteroaromatic substrates bearing a thiophene- or pyridine residue were tolerated in this hydroalkynylation reaction. Although four different products are possible for the hydroalkynylation of unsymmetrical substituted alkynes, only the *Z*-configured regioisomers **6** and **7** were observed, while the isomers **8** and **9** (compare Scheme 2) had not been mentioned. A plausible mechanism was postulated, which is in accordance with the already discussed mechanism shown in Scheme 12, just differing in the second coordination which had to be substituted with an internal alkyne instead of a terminal alkyne. Because of the competing reactions, hydroalkynylation vs. homodimerization, the hydroalkynylation of internal alkynes seemed to be faster than the homodimerization of the silyl-substituted alkyne (Sakurada et al., 2013).

## 4 Nickel-Catalyzed Hydroalkynylation Reactions

Neither nickel-catalyzed homodimerization nor cross-dimerization toward 1,3-enynes of type **2**–**4** has been described in the literature yet, due to the high reactivity of the explored catalysts toward the primary enyne products. However, some examples of cross-addition of terminal silyl-substituted alkynes to internal alkynes have been reported.

**SCHEME 22:**
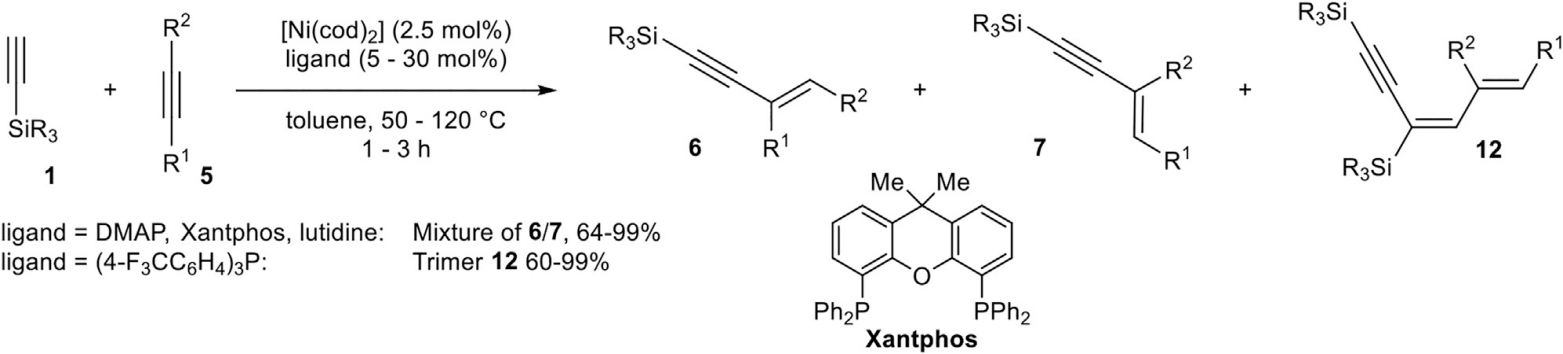
Nickel-catalyzed hydroalkynylation between internal alkynes and silyl-substituted alkynes (Matsuyama et al., 2008).

Miura developed a catalyst system consisting of [Ni(cod)_2_] and 4-(dimethylamino)pyridine (DMAP), Xantphos, and 2,6-lutidine or tris(4-fluoromethylphenyl)phosphine as ligand in toluene, which catalyzed the hydroalkynylation between terminal silyl-substituted alkynes **1** and symmetrical and unsymmetrical substituted activated alkynes (Scheme 22) (Matsuyama et al., 2008).

DMAP as the ligand led to the selective formation of product **6** in the case of diphenyl acetylene as acceptor-alkyne **5**. In case of the unsymmetrical substituted alkyne 3-phenyl-prop-2-yn-1-ol the choice of ligand had a crucial effect on the regioselectivity of the hydroalkynylation. DMAP gave predominantly the regioisomer **6** (with R^1^ = Ph), while 2,6-lutidine inverted the regioselectivity toward the regioisomer **7**. Xantphos as the ligand increased the yield of **6** in comparison to DMAP. Furthermore, tris(4-trifluorophenyl)phosphine as the ligand changed the reaction pathway toward 1:2 hydroalkynylation products of type **12** (Matsuyama et al., 2008).

**SCHEME 23:**
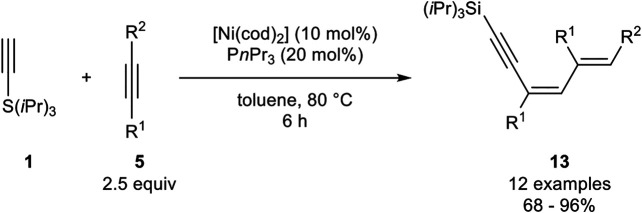
Nickel-catalyzed 1:2 trimerization between silyl-substituted alkynes and internal alkynes (Ogata et al., 2009a).

This type of trimerization was also investigated by Fukuzawa, who investigated a nickel catalyst system, consisting of [Ni(cod)_2_] and triphenylphosphine as ligands in toluene at 80°C (Scheme 23) (Ogata et al., 2009a).

In contrast to the catalyst system developed by Miura, this catalyst system led to the formation of the 1:2 trimer where one silyl-substituted alkyne reacts with two equivalents of the internal alkyne to give the 1,3,5-dienynes **13**. The authors altered the residues of the used internal alkyne, where besides alkyl substituents, ethers as well as aryl residues were tolerated. However, diphenyl acetylene did not undergo this 1:2 trimerization. Unsymmetrical substituted alkynes with aryl moieties gave only one isomer, in which the new carbon-carbon bond is formed at the carbon-atom with no aryl residue (*R* ^2^ = Ar) (Ogata et al., 2009a).

**SCHEME 24:**
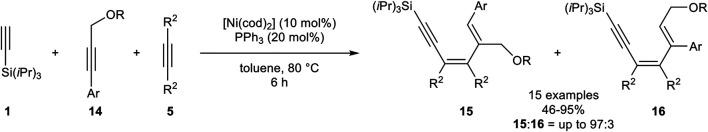
Nickel-catalyzed linear trimerization between silyl-substituted alkynes, propargylic ethers, and internal alkynes (Ogata et al., 2009b).

Next, Fukuzawa investigated a highly chemoselective linear trimerization utilizing three different alkynes as substrates. Instead of tri*-n-*butylphosphine, triphenylphosphine showed the best results. As alkynes, triisopropylsilyl acetylene **1**) as the terminal alkyne, an aryl-substituted propargylic ether **14,** and 3-hexyne **5**) were used (Scheme 24) (Ogata et al., 2009b).

This catalyst system gave high regioselectivities between product **15** and product **16** (**15**:**16** = up to 93:7), with isolated yields between 74 and 92%. Different substitution patterns of the aryl-moiety were tested, where *para*-substituted aryl residues gave significantly higher yields than *ortho*- or *meta-*substituted residues. Also, different ethers were investigated, showing no significant influence either on the yield or on the regioselectivity. Furthermore, the chain length of the aliphatic internal alkyne was altered. While 2-octyne as internal alkyne **5** resulted in a decreased yield, the use of 2-butyne in a large excess of 5.00 equivalents gave the desired product **15** in 95% yield (**15**:**16** = 92:8). Cyclohexyl substituted propargyl ether **14** underwent the trimerization toward **15** in a good yield of 68% and excellent stereoselectivity (**15**:**16** > 99:1). Other aliphatic residues, e.g., *n*-hexyl, resulted in a complex mixture (Ogata et al., 2009b).

## 5 Conclusion

In recent years, highly chemo-, regio-, and stereoselective dimerization protocols with iron or cobalt complexes as catalysts toward the *head-to-head* dimerization products of type **2** and **3**, as well as the *head-to-tail* dimerization products of type **4,** have been developed. While cobalt catalysts seem to be superior in comparison to iron catalysts for the *E*-selective (cross-) dimerization of terminal alkynes, iron catalysts gave better results in the *Z*-selective transformations. *Head-to-tail* homodimerization has been well studied for piano-stool type iron(II) catalysts, which are also able to cross-dimerize alkynes if a propargylic alcohol or propargylic amine is used as the donor-alkyne. A very versatile protocol has been described using a cobalt(II)-TriPhos^tBu^ catalyst with a high chemoselectivity, which is determined by the acidity of the used alkynes. The hydroalkynylation of internal alkynes was investigated for cobalt phosphine complexes using silyl-substituted alkynes as donors. Unfortunately, nickel catalysts have not been described for the selective dimerization of two terminal alkynes. However, hydroalkynylation between internal alkynes and silyl-substituted acetylenes has been developed. Due to their high reactivity, the nickel-catalyzed hydroalkynylation could be expanded toward linear 1:2 trimerization or a high chemoselective trimerization, utilizing three different alkynes.

Nevertheless, some drawbacks have to be mentioned; 1) aliphatic alkynes are mostly not tolerated in *head-to-head* homodimerization, leading to no conversion or higher oligomers. In *head-to-head* cross-dimerization, aliphatic alkynes are only tolerated as the acceptor-alkyne or as the donor-alkyne, when bulky substituents are next to the triple bond; 2) for the cross-dimerization toward the *head-to-head* products only bulky donor-alkynes such as silyl-substituted alkynes can be used without the loss of chemoselectivity; 3) the hydroalkynylation of internal alkynes always needs a silyl-substituted acetylene to give high yields of the desired product, while the regioselectivity of the possible isomers is not controlled effectively in cobalt-catalyzed reactions.
